# Postoperative aortic injury caused by a staple line formed during wedge resection of the lung

**DOI:** 10.1093/icvts/ivac275

**Published:** 2022-11-24

**Authors:** Masayuki Yamaji, Motoki Yano, Sawako Okamaoto, Takayuki Fukui

**Affiliations:** Division of Chest Surgery, Department of Surgery, Aichi Medical University, Nagakute, Japan; Surgical Oncology Center, Oncology Center, Aichi Medical University Hospital, Nagakute, Japan; Division of Chest Surgery, Department of Surgery, Aichi Medical University, Nagakute, Japan; Division of Chest Surgery, Department of Surgery, Aichi Medical University, Nagakute, Japan

**Keywords:** Aortic injury, Automatic stapling, Adverse event

## Abstract

We report a case of aortic perforation caused by the staple line formed during a wedge resection for lung cancer. Six hours after an uneventful wedge resection, sudden frank drainage of blood from the chest tube occurred. A reoperation was performed, and we found bleeding from the aorta. After suturing the bleeding spot on the aorta, we found that the stapling line of the lung rode on the aorta with longitudinal contact. We speculated that the stapling line scratched the aorta in synchrony with the patient's breathing and injured the aorta.

## INTRODUCTION

We report a case with postoperative aortic injury caused by the staple line formed during a wedge resection of lung cancer. Postoperative aortic injury is rare. It is a critical condition, and it is usually difficult to save the patient.

## CASE

**Figure 1:**
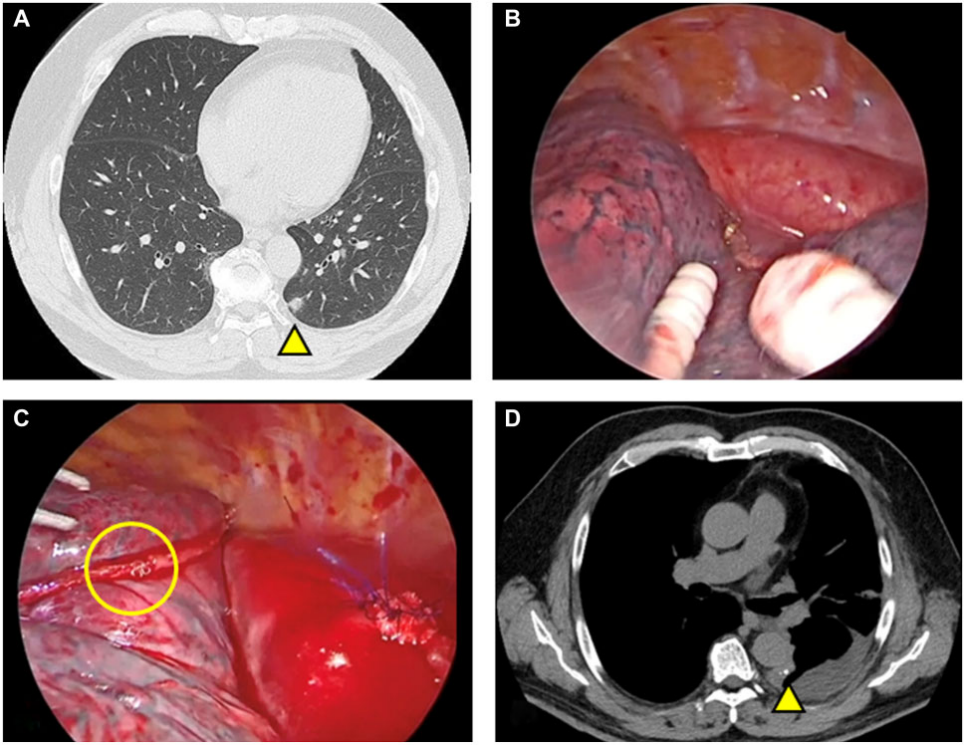
Preoperative and postoperative chest computed tomography and intraoperative findings. (**A**) Computed tomography revealed a lung nodule in the lower lobe of the left lung (yellow triangle). (**B**) Intraoperative findings of the first operation: No instrument-related injury to the aorta was found. (**C**) Intraoperative findings from the second operation comprised a wedge resection and the sutured aorta. The formation of an incomplete beta loop and damaged staples were not found on the stapling line, though a few staples on the stapling line were intertwined. (**D**) Postoperative chest computed tomography: The staple line contacts the aorta longitudinally.

**Figure 2:**
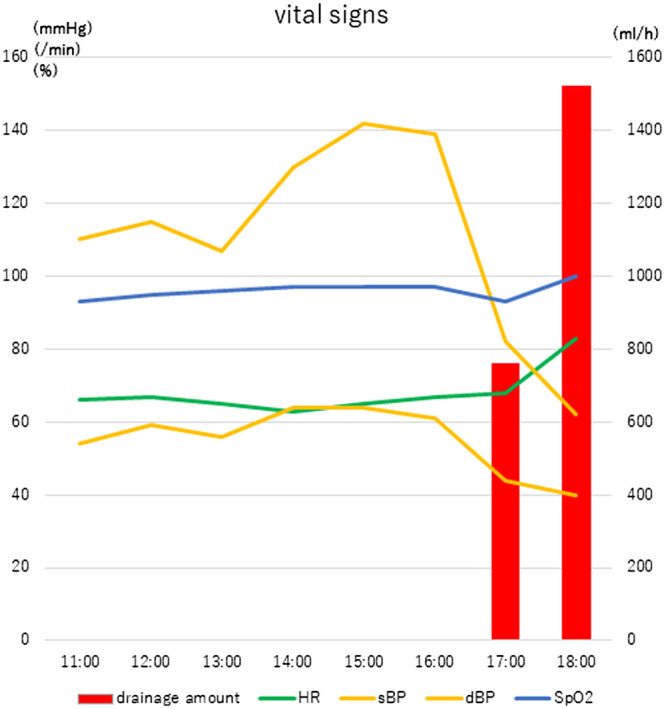
Postoperative vital signs while the patient was in the intensive care unit. The patient's vital signs remained stable for 6 h postoperatively. Then, suddenly, fresh blood appeared in the fluid drained from the wound and his blood pressure dropped. HR: heart rate; dBP: diastolic blood pressure; sBP: systolic blood pressure; S_p_O_2:_ oxygen nadir.

A 61-year-old male presented to our department with bilateral small nodules that were suspected to be bilateral multiple primary lung cancers. We first planned a wedge resection of the lower lobe of the left lung (Fig. 1A) and then a lower lobectomy of the right lung laterally. During the first operation, a nodule was found in S10 of the left lower lobe with pleural indentation. Following resection of the pulmonary ligament, a wedge resection of the lung was performed (Fig. 1B). We noted through touch the fragility of the tissues throughout the operation. The patient's postoperative condition was stable, but he complained of back pain. Pleural effusion was almost nonexistent for 6 h after the operation, but sanguineous drainage appeared suddenly, and his blood pressure dropped (Fig. 2). We hypothesized that the postoperative bleeding originated from an intercostal artery at the minithoracotomy.

We started the second operation promptly. The thoracic cavity was filled with haematomas (Video 1). Following removal of the haematomas, we found a mediastinal haematoma and bleeding from a defect in the mediastinal pleura. We inserted a finger through the defect and discovered bleeding from the aorta. We sutured the bleeding spot on the aorta. Subsequently, we found that the stapling line of the wedge resection of the lung rode longitudinally on the aorta. We also found a few staples on the stapling line that were intertwined, but we did not find any staples involved in an incomplete beta loop formation or that were destroyed (Fig. 1C). We covered the staple line with bioabsorbable polyglycolic acid fabric and fibrin glue. On postoperative day 8, the patient was discharged (Fig. 1D).

## DISCUSSION AND CONCLUSION

We have previously reported adverse events from stapling in thoracic surgical procedures. In 10,908 lung tissue staplings, 81 adverse events (0.74%) occurred [1]. Only 1 case was reported with arterial bleeding (0.01%) from the intercostal artery [2]. However, we could not find any cases with aortic injury caused by the staple line formed during wedge resection of the lung. In abdominal surgery, we found a few reports of cases with an aortoenteric fistula caused by the staple line [3]. Honda *et al.* suggested the importance of covering seromuscular sutures to prevent contact with the aorta [3].

We looked at the video of the first operation and confirmed the absence of any instrumental injury to the aorta (Fig. 1B). We found during the second operation that the stapling line rode on the aorta with longitudinal contact. We speculated that the stapling line scratched the aorta in synchrony with the patient's breathing and finally injured the aorta even though the staples were not damaged. Even so, we could not deny the possibility of an undetected minor injury caused by the instruments. When we made the skin incision or dissected the connective tissues at the thoracic ports, we could feel the fragility of the tissue by touch. The ability to sense this fragility by touch may be important to predict postoperative tissue injury.

We emphasize the importance of checking the integrity of every staple line and removing any sharp edges that emerge from the staple line and/or of covering the staple line in the lung that rides on the aorta. Cooperation and communication among staff in the operating room and the intensive care unit, the anaesthesiologist and the cardiac surgeons are also essential for preventing injury to the tissues.

By exhibiting careful attention to detail, we were able to save this patient who developed a rare postoperative aortic injury caused by the staple line formed during wedge resection of lung cancer.
